# Addressing the challenges of prevention and control of West Nile virus in Africa: A correspondence

**DOI:** 10.1097/JS9.0000000000000188

**Published:** 2023-01-24

**Authors:** Bezawit K. Bekele, Olivier Uwishema, Abubakar Nazir, Ikshwaki Kaushik, Jack Wellington

**Affiliations:** a Oli Health Magazine Organization, Research and Education, Kigali, Rwanda; b Addis Ababa University, Addis Ababa, Ethiopia; c Clinton Global Initiative University, New York, USA; d Faculty of Medicine, Karadeniz Technical University, Trabzon, Turkey; e Department of Medicine, King Edward Medical University, Lahore, Pakistan; f David Tvildiani Medical University, Tbilisi, Georgia; g Faculty of Medicine, Cardiff University School of Medicine, Cardiff University, Cardiff, UK

HIGHLIGHTS

- West Nile virus (WNV) is primarily diagnosed via serological testing, detecting antibodies against WNV in the blood, and the cerebrospinal fluid. Practically, the majority of patients are no longer viraemic at the time of symptom onset due to brief viraemic phases and the low viral load present in the blood of affected persons.
- The African continent’s abundance of ornithophilic mosquitoes and wild birds provides an ideal environment for the spread of WNV to horses and other susceptible animals. Migration pathways play a significant part in the spread of WNV.
- Disease-preventative measures frequently come into effect following established human infection; measures aimed against the mosquito population may be warranted.

West Nile virus (WNV), a mosquito-borne flavivirus first discovered in Uganda in 1937^1^. Transmission of WNV concerns mosquitoes, primarily *Culex* species, which feed on birds having elevated levels of the virus in the blood^2^. Subsequently, mosquitoes become infected with WNV and then transmit said virus to susceptible vertebrates they may feed upon. These include humans and horses, which are regarded as dead-end hosts^3^.

Among infected humans, 80% are asymptomatic, of which most exhibit self-limiting clinical manifestations, such as acute fever, headache, rash, fatigue, gastrointestinal symptoms, myalgia, and weakness^3^. Less than 1% of WNV-infected persons develop neuroinvasive disease-causing meningoencephalitis or acute flaccid paralysis^4^. Neuroinvasive disease, in general, is associated with significant long-term morbidity and was found to have a fatality rate of 10%^4^.

The most popular method for determining WNV infection is serological testing. One of the most used approaches for identifying WNV disease is the detection of WNV antibodies in serum and cerebrospinal fluid^5^.

Currently, WNV infection has no cure. Due to the absence of vaccines for the prevention of the disease, infection prevention by mosquito bite protection is thus a vital public health strategy. In addition, once the disease presents symptomatically because of the lack of effective antiviral therapy, management of WNV infection is mostly supportive.

At first, it was believed that WNV could only be discovered in sub-Saharan African (SSA) nations. The WNV was later discovered in Europe and the Americas^3^. Migratory birds are often cited as a means of translocation between said continents^6^. While the burden of the disease has been well documented in North America and Europe, the true magnitude of the WNV burden in the African continent is poorly understood due to poor surveillance and diagnostic limitations. However, evidence from seroprevalence studies and disease reports indicate that WNV is endemic all over sub-Saharan African wherever there is an abundant population of susceptible mosquitoes^3^. In addition, WNV is prevalent in nations including north-western Africa. This infection is cyclical and most likely influenced by the population’s immunological level, the vector’s abundance, the availability of amplifying hosts, and environmental conditions^3^.

Due to increasing morbidity and death in humans, birds, and horses, WNV’s continuous spread and reappearance in African countries pose concerns to public health and veterinary care^7^. The African continent’s abundance of ornithophilic mosquitoes and wild birds provides an ideal environment for the spread of WNV to horses and other susceptible animals. Migration pathways play a significant part in the spread of WNV^8^. Migratory birds are abundant in Africa^8^. Climate change has had a significant impact on the vectors that spread WNV, and other vector-borne illnesses, from endemic to nonendemic areas^9^. Human and animal health are significantly harmed by climate change, especially in developing nations^9^.

Given that there is no WNV vaccine, it is essential to understand how infection is acquired. Public health initiatives can help prevent WNV. There are two ways to stop further outbreaks of WNV postinfection onset. Individuals should advocate and employ personal protective measures, such as removing standing water sources at home, ensuring doors and windows have well-fitting screens, staying inside between dusk and dawn, dressing in long pants and sleeves, and *N*,*N*-Diethyl-*meta*-toluamide insect repellent when outside. These measures should be emphasized to individuals over the age of 50 and those with chronic medical conditions. To prevent sickness, the second option entails local health organizations working alongside state health agencies and the Centers for Disease Control and Prevention. Local health organizations must conduct active surveillance to identify and track the prevalence of sickness in the neighborhood. They should maintain expert knowledge of WNV epidemiology and sickness patterns when developing control methods. Public health organizations should inform politicians and the general public. In urban areas, monitoring involves setting up mosquito traps in strategic locations and routinely testing the captured mosquitos for WNV infection. Rising rates of mosquito infection predict rising rates of human infection.

The actions listed below ought to be performed if WNV is found in a particular area: individuals should spend as little time outside as possible between sunset and sunrise, wear shoes, socks, long pants, and a long-sleeved shirt when spending extended periods outside or when mosquitoes are most active. The use of mosquito repellent with *N*,*N*-diethyl-*meta*-toluamide, picaridin, oil of lemon eucalyptus, or ethyl butyl acetylamino propionate (IR3535) should be applied as directed. Further, the removal of any standing water around homes and property to aid the reduction of mosquito populations alongside discarded tires from properties should be employed.

We call on the African leaders and concerned stakeholders to implement One Health action in their efforts to control WNV in Africa. A multidisciplinary framework targeted at veterinary and human health will yield the best result. To boost trust and encourage adherence of the general public to WNV preventative measures, good policies and participation of the public in policymaking and health literacy promotion should be employed.

Further research should be conducted to determine the burden of WNV in African countries with no data on WNV. This will enable necessary control and preventive surveillance, interventions, and policies to be instituted at local, regional, and national levels. Concerned stakeholders should intensify their efforts to develop drugs and human vaccines for WNV. Private entities and Africans with high net worth should fund human capacity building, infrastructure, and technological development, and promote research.

Amidst the coronavirus disease pandemic, it is crucial to have a clear distinction between the two viral pathogens. Below is a table highlighting the differences between the two viruses ( Table 1 and Fig. 1).

**Table 1:** Comparison between COVID-19 and WNV^10^.

	COVID-19	WNV
Most common symptoms	Fever, cough, and dyspnea	Fever, headache, tiredness, body ache, nausea, and vomiting
Similar symptoms	Headache, fever, nausea and vomiting, fatigue, and sore throat	
Unique symptoms	Anosmia and/or taste, rhinorrhoea, and congestion	Skin rash (on the trunk) and swollen lymph nodes
Less common symptoms	Conjunctivitis and rash on the skin	Neuroinvasive disease, headache, fever, neck stiffness, disorientation, tremors, coma, convulsions, muscle weakness, and paralysis
Incubation period	2–14 days	3–14 days
Mortality rate	~2.1%	~0.03–0.15%
Way of transmission	Respiratory droplets	Infected mosquito bites, contact with infected animal blood or tissues, and mother to child
Asymptomatic	40–45%	80%

COVID-19 indicates coronavirus disease 2019; WNV, West Nile virus.

**Figure 1:**
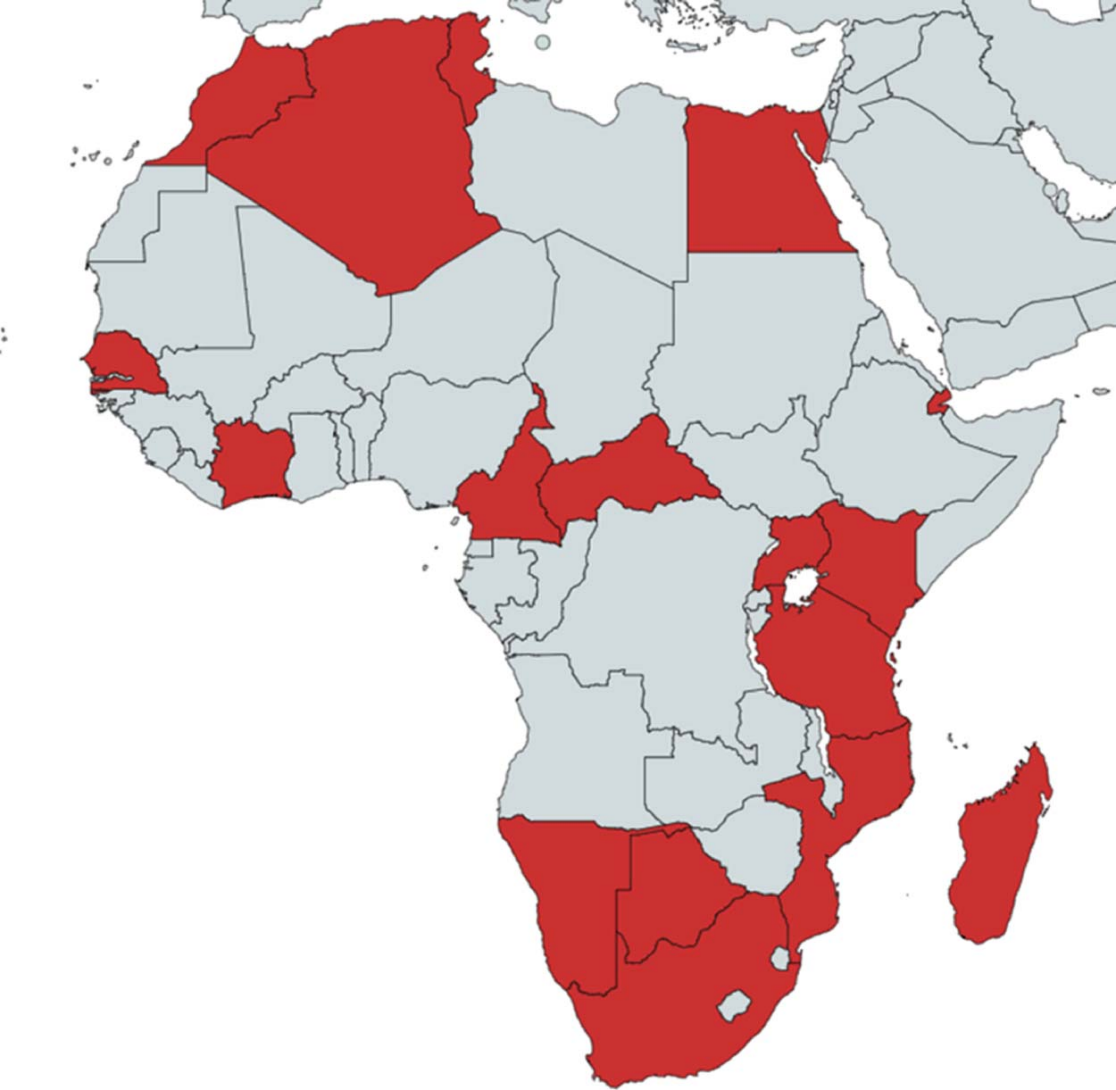
Map: African countries in which lineages of West Nile virus were confirmed (https://doi.org/10.1371/journal.pntd.0010075 this is the reference for the countries on the map shown above).

## Ethical approval

Not applicable.

## Sources of funding

Not applicable.

## Author contribution

O.U.: conceptualization, project administration, and writing – review and designing. All authors were involved in manuscript writing, data collection and assembly, and final approval of the manuscript.

## Conflicts of interest disclosure

There are no conflicts of interest.

## Guarantor

A. Nazir; affiliation: Oli Health Magazine and Organization, Kigali, Rwanda; E-mail: abu07909@gmail.com; ORCID ID: 0000-0002-6650-6982.
